# Involvement of median preoptic nucleus and medullary noradrenergic neurons in cardiovascular and sympathetic responses of hemorrhagic rats

**DOI:** 10.1038/s41598-018-29310-z

**Published:** 2018-07-26

**Authors:** Lara Marques Naves, Stefanne Madalena Marques, Aline Andrade Mourão, James Oluwagbamigbe Fajemiroye, Carlos Henrique Xavier, Carlos Henrique de Castro, Ana Cristina Silva Rebelo, Daniel Alves Rosa, Rodrigo Mello Gomes, Eduardo Colombari, Gustavo Rodrigues Pedrino

**Affiliations:** 1 Departamento de Ciências Fisiológicas, Instituto de Ciências Biológicas, Universidade Federal de Goiás, Estrada do Campus, s/n, 74690-900 Goiânia, GO Brazil; 2 Faculdade de Farmácia, Universidade Federal de Goiás, Goiânia, Goiás Brazil; 3 Departamento de Morfologia, Instituto de Ciências Biológicas, Universidade Federal de Goiás, Estrada do Campus, s/n, 74690-900 Goiânia, GO Brazil; 4 Departamento de Fisiologia e Patologia, Faculdade de odontologia, Universidade Estadual Paulista, Araraquara, São Paulo Brazil

## Abstract

The infusion of hypertonic saline solution (HSS) is known to be beneficial to the treatment of hypovolemic hemorrhage (HH). The central mechanism of HSS-induced cardiovascular and autonomic recovery of animals subjected to HH remains unclear. Hence, the present study evaluated the involvement of median preoptic nucleus (MnPO) and medullary noradrenergic neurons (A1 and A2) in HSS-induced cardiovascular and sympathetic responses in hemorrhagic rats. The wistar rats were subjected to specific lesion of noradrenergic neurons through the nanoinjections of anti-DβH-saporin into caudal ventrolateral medulla (A1 neurons) and nucleus of the solitary tract (A2 neurons). After recovery, mean arterial pressure (MAP) and renal sympathetic nervous activity were recorded. The HH was performed through blood withdrawal until a MAP of 60 mmHg was attained. In sham rats, HSS infusion (3M NaCl) reestablished MAP without change in HH-induced sympathoinhibition. The muscimol (agonist of GABA_A_ receptor) was nanoinjected in MnPO during HH and MnPO inhibition abolished the recovery of MAP and HSS-induced sympathoinhibition. Simultaneous lesions of A1 and A2 abolished MAP restoration and sympathoinhibition after HSS infusion. These results suggest that the recovery of MAP and HSS-induced sympathoinhibition in hemorrhaged rats depend on intact neural projections from A1 and A2 to MnPO.

## Introduction

Hypovolemic hemorrhage (HH) is often characterized by the loss of a large blood volume as a result of serious injury. The occurrence of HH incapacitates the body and prevent adequate blood perfusion to vital organs and systems^1^. The hypoperfusion of tissue constitutes a risk factor to many cardiovascular complications, diseases and high mortality rates^2,3^.

Although several therapies have been developed to treat HH^4,5^, the administration of hypertonic saline solution (HSS) has attracted more interest because it produces cardiovascular recovery in smaller volumes^6^. Previous evidence suggests that the hemodynamic effects of HSS are not solely attributed to the plasma volume expansion^7^. According to Costa *et al*.^8^ the restoration of blood pressure during hemorrhage following HSS infusion can be blocked by non-selective denervation of baroreceptors^8^ and inactivation of carotid chemoreceptors^9^. Although, these results suggest the participation of peripheral neural afferents in HSS-induced hemodynamic improvement, the centrally mediated regulation remains unclear.

The peripheral neural afferents, with projections to the nucleus of the solitary tract (NTS, A2 neuronal catecholaminergic cluster), often detect and integrate changes in the composition and circulating volume^10^. The NTS conducts these changes to medullary, pontine and hypothalamic regions that are responsible for autonomic, cardiovascular and humoral adjustments^11–15^. The caudal ventrolateral medulla (CVLM, A1 neuronal catecholaminergic cluster) and the median preoptic nucleus (MnPO) which have been identified in these regions are known to play important roles. The anteroventral third ventricle region (AV3V) region of MnPO do not only acts as an information integration center but also maintains cardiovascular and electrolyte balance^16–19^. Previous studies have revealed direct and indirect projections of the A1 and A2 neuronal clusters to the AV3V, organum vasculosum of the lamina terminalis (OVLT) and the medial portions of the periventricular pre-optic nucleus^15,20,21^.

The vital role of MnPO, A1 and A2 in the regulation of body fluid, autonomic and cardiovascular changes makes the investigation of their integrity in HSS-induced recovery important towards the understanding of the neuronal mechanism underlining HH. In this manner, it can be hypothesized that these regions participate in neuronal mechanisms that regulate reflex sodium overload-responses during hypovolemia. Hence, this study evaluated the involvement of MnPO, A1 and A2 noradrenergic neurons clusters in HSS-induced cardiovascular and sympathetic compensatory responses in HH rats.

## Methods

### Animal model

Adult Wistar rats (8–10 weeks, 280–320 g) provided by the central animal facility of the Universidade Federal de Goiás (UFG) were used. The rats were housed in the Department of Physiological Sciences (DCiF) under light and temperature-controlled conditions (12-h light–dark cycle, 22–24 °C) with free access to water and food. All the experimental procedures were carried out according to the rules and guidelines for care and use of laboratory animals as approved by the ethics committee of the UFG (protocol number 034/12).

### Nanoinjection into MnPO and pharmacological inhibition

The muscimol or saline nanoinjected into MnPO at an interval of 10 minutes after HH. The nanoinjections were performed with a glass micropipette coupled to a manual pressure system. The parietal and frontal bone were partly removed to perform the nanoinjections. The bregma was located and the coordinates were recorded for positioning of the glass pipette. The injected volume of each solution was controlled by meniscus displacement of the solution in the micropipette through a surgical microscope equipped with an ocular lens with calibrated reticulum.

In order to achieve pharmacological inhibition, the animals received nanoinjections of Muscimol (GABAA receptor agonist, 4 mM, 100 nL, Sigma-Aldrich, St. Louis, MO, USA) in the MnPO. The sham group received nanoinjections of saline (NaCl, 0.15 M, 100 nL) were performed in the MnPO. At the end of the experiments, the Evans blue dye (4%, 100 nL, Sigma-Aldrich, St. Louis, MO, USA) was nanoinjected into the same region for histological confirmation of the sites. The Paxinos & Watson atlas^16^ was used to determine the coordinates. For all nanoinjections into the MnPO, a glass micropipette was positioned at 0.6 mm rostral to bregma, 0.0 mm lateral to midline, and 7.2 mm below dura mater^17^. Only animals whose nanoinjections were restricted to the MnPO region were considered for analysis.

### Nanoinjections into the CVLM and NTS

The animals belonging to the A1 and A2 neuronal lesion protocol were anesthetized with a mixture of ketamine (10%, 1 mL·kg^−1^, i.p., Syntec, Santana de Parnaíba, SP, Brazil) and Xylazine (2%, 0.7 mL·kg^−1^, i.p., Syntec Santana de Parnaíba, SP, Brazil) on a stereotaxic apparatus (Insight Ltda., Ribeirão Preto, SP, Brazil) with incisor bar 11 mm below the interaural line. An incision was made in the posterior inferior region of the head, to expose the occipital bone and the atlanto-occipital membrane. The occipital bone was removed and the *calamus scriptorius* was used as a reference point for the stereotactic coordinates. In order to achieve A1 and/or A2 neuronal lesions, the anti-dopamine-β-hydroxylase-saporin complex (anti-DβH-saporin, 100 nL, 0.105 ng·nL^−1^, Advanced Targeting Systems, San Diego, CA, USA) was nanoinjected into the CVLM and NTS region, respectively. In sham groups, the equimolar of Saporin (100 nL, 0.022 ng·nL^−1^, Advanced Targeting Systems, San Diego, CA, USA) was nanoinjected into the same site. For all nanoinjections into the CVLM, a glass micropipette was positioned at 0.3 mm rostral and 0.2 mm caudal from the *calamus scriptorius*, 1.8 mm lateral from the mid-line, and 1.8 mm ventral from the dorsal surface. For all nanoinjections into the NTS, a glass micropipette was positioned at 0.0 and −0.5 mm caudal to *calamus scriptorius*, 0.0 mm lateral from the mid-line and 0.3 mm ventral from the dorsal surface. These coordinates were based on the region of the CVLM and NTS consisting of the A1 and A2 neurons groups, respectively^18,19^.

At the end of the central nanoinjections, the incision was sutured with surgical line prior to the administration of analgesic (Flunixin, 0.02 mL·kg^−1^, i.m., CHEMITEC, Brazil, SP). The animals were later housed during 20 days with free access to water and food to ensure surgical recovery and lesions establishment.

### Surgical procedures

The animals were subjected to anesthetic induction through the administration of halothane (2%, Tanohalo, Cristália, Itapira, SP, Brazil) in 100% O_2_ prior to the catheterization of femoral artery and vein. After vein catheterization, anesthesia was maintained by administration of urethane (1.2 g·kg^−1^, i.v., Sigma-Aldrich, St. Louis, MO, USA). Additional catheter was inserted into the right carotid artery to withdraw blood during HH. Tracheostomy was performed to reduce airway resistance. In order to record renal sympathetic nervous activity (RSNA), left renal nerve was isolated and positioned on silver bipolar electrodes. The body temperature was maintained between 36 °C and 37 °C with a thermostatically controlled heated table.

### Hypovolemic hemorrhage and sodium overload

The HH was induced through blood withdrawal over 20 minutes until the values of MAP reached 60 mmHg. Sodium overload was achieved during 90 seconds of HSS (3 M NaCl, 1.8 mL·kg^−1^, Sigma-Aldrich, St. Louis, MO, USA) infusion.

### Recording of blood pressure and heart rate

The pulsatile arterial pressure (PAP) signal was obtained by connecting the femoral artery catheter to a pressure transducer (MLT0699, ADInstruments Bella Vista, Australia) coupled to an amplifier (Bridge Amp, FE 221, ADInstruments, Bella Vista, Australia). Data were digitized at a frequency of 2000 samples·s^−1^ using a digital analog converter (PowerLab 4/25, ML845, ADInstruments, Bella Vista, Australia). The MAP was calculated from the PAP signal integral (LabChart 7, v7.3.7, ADInstruments, Bella Vista, Australia). Heart rate (HR) was calculated as the instantaneous frequency of the PAP signal (LabChart 7, v7.3.7, ADInstruments, Bella Vista, Australia).

### Recording of renal sympathetic nerve activity (RSNA)

The RSNA was recorded through the left renal nerve with bipolar silver electrodes. The renal nerve was located, dissected and covered with mineral oil (Nujol, Schering-Plough, São Paulo, SP, Brazil) prior to the placement of electrodes for recording. The signals were obtained using a high-impedance probe connected to the amplifier (P511, Grass Instruments, Quincy, MA, USA). The signal was amplified 20.000 times, digitized and band-pass filtered (30–1000 Hz). The nerve signal was recorded continuously (2000 samples · s^−1^, PowerLab 4/25, ML845, ADInstruments, Bella Vista, Austrália) rectified and integrated at 1 s intervals using LabChart software (v.7.3.7., ADInstruments, Bella Vista, Austrália). At the end of each experiment, ganglionic blocker hexamethonium (30 mg·kg^−1^, b.wt., i.v., Sigma–Aldrich, St. Louis, MO, USA) was administered to determine the background noise. The level of RSNA was expressed as a percentage of baseline after subtraction of the noise. Functionality of baroreceptor reflexes in baseline period was also evaluated. For this, fast Fourier transform was performed on 60 s windows after the AP and SNA signals were configured and sampled at 1000 Hz. The frequency resolution of the spectra was 0.2 Hz/bin. The spectra in this paper show frequencies between 0 and 10 Hz. The same windows were used for coherence analysis by using coherence script for Spikes 2 software as described^22^.

### Recording of blood flow

The renal blood flow (RBF) and aortic blood flow (ABF) baseline were recorded through a miniature probe that was placed around the left renal artery and abdominal aorta. Miniature probes were connected to a T206 flowmeter (Transonic Systems, Inc., Ithaca, NY, USA) which determines the flow rate in absolute values (ml·min^−1^). The signals obtained were transferred to the acquisition and data analysis software (PowerLab 4/25, ML845, ADInstruments, Colorado Springs, CO, USA). Data was digitized at a sampling frequency of 1000 samples per second. The hind limb blood flow (HBF) was calculated by using the following equation: (ABF) − (2 x RBF).

### Immunohistochemistry

The immunohistochemistry technique was employed to label medullary sections with positive tyrosine hydroxylase cells (TH positive) to determine the extent of A2 and A1 neuronal lesion. At the end of the experiments, animals were perfused with saline (0.15 M NaCl, 200 mL) followed by paraformaldehyde solution (0.2 M, 500 mL, Sigma-Aldrich, St. Louis, MO, USA) in sodium phosphate buffer (pH 7.4). The brainstem was cut in coronal sections of 40 µm thickness with freezing microtome (CM1860, Leica, Deutsch, Germany). The brainstem was later collected (in 4 serially adjacent sets) and stored in sodium phosphate-buffered saline (0.02 M, PBS, Sigma-Aldrich, St. Louis, MO, USA, pH 7.4). The brain of each rat was removed and post fixed in paraformaldehyde (0.2 M, 30 mL) solution for 1–2 h and then dehydrate in sucrose solution (0.8 M, 30 mL, Sigma-Aldrich, St. Louis, MO, USA).

Each of the fourth brainstem section were washed in ImmunoBuffer (IB, Triton 0.3% in phosphate buffer saline/PBS, Sigma-Aldrich, St. Louis, MO, EUA) followed by a 30-min incubation in 2% normal horse serum (Vector Laboratories Inc., Burlingame, CA, USA) in IB. The sections were incubated overnight with mouse monoclonal antibody (1:2000 dilution, Immuno Star Inc., Hudson, WI, USA) with 2% normal horse serum in IB, followed by an overnight incubation with biotinylated horse anti-mouse IgG (1:500 dilution, Vector Laboratories Inc., Burlingame, CA, USA). After these incubations, the sections were processed with the avidin-biotin procedure using Elite Vectastain reagents (Vector Laboratories Inc., Burlingame, CA, USA). The diaminobenzidine (DAB) was used to produce a brown cytoplasmic TH reaction product.

### Neuronal count

The TH-labeled neurons were counted in each of the fourth section of medulla (40 of each 160 μm). All cells with cell body and at least one dendrite or axon in the VLM (A1/C1 neuronal clusters) and NTS (A2/C2neuronal clusters) were counted bilaterally to quantify the extent of anti-DβH-saporin induced lesion. The neurons (at the magnification 200x) were counted with the aid of an optical microscope (Leica DM500, Leica Microsystems, SP, Brazil) coupled to a digital image acquisition system (Leica Application Suite, V.3.10, Leica Microsystems, Brazil).

### Statistical analysis

The statistical analyses were performed using the GraphPad Prism software (v 5.01). Cardiovascular and autonomic parameters were expressed as mean ± standard error of the mean (SEM). The MAP, HR and RSNA variations were analyzed using two-way analysis of variance (ANOVA two way) followed by Tukey’s post hoc test. The value of p < 0.05 was considered a significant difference.

The cell count was expressed as mean ± SEM. The number of cells counted for each section was compared by one-way analysis of variance (ANOVA one way) followed by Tukey’s post hoc test. The total cell counts for groups A1, C1, A2, and C2 were compared between groups by Student’s t-test. Value of p < 0.05 was considered to be significantly different.

### Data availability statements

The datasets generated during and/or analyzed during the current study are available from the corresponding author on reasonable request.

## Results

### Effects of pharmacological inhibition of MnPO on HSS-induced cardiovascular and sympathetic responses

**Figure 1:**
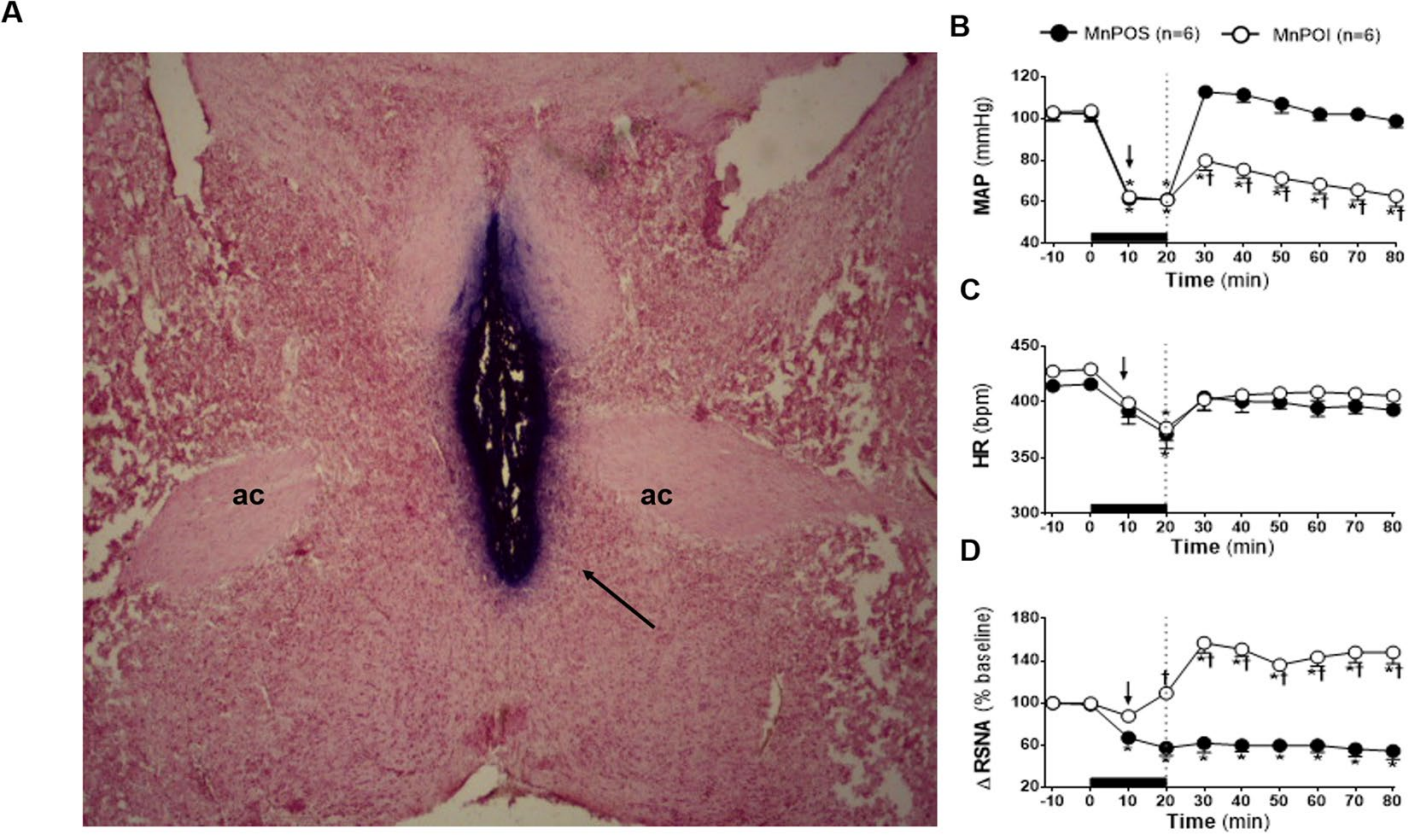
**(A)** Representative photomicrograph of coronal section of the hypothalamus (40 μm) showing a typical site of nanoinjection in the median preoptic nucleus (MnPO). The arrow indicates the marking of the MnPO with evans blue dye 4%. AC: anterior commissure. The scale bar is equal to 500 µm. **(B)** Effects of MnPO pharmacological inhibition on changes in Mean arterial pressure (MAP), (**C**) heart rate (HR) and **(D)** integral of renal sympathetic nervous activity (∫RSNA) variations in the rats submitted to hypovolemic hemorrhagic in the MnPO Sham (MnPOS; n = 6) and MnPO Inhibition (MnPOI; n = 6) groups. Values were expressed as means ± standard error of the mean (S.E.M). *Different from time 0; ^†^different from sham group with p < 0.05. The black bar represents the hemorrhage period. The dashed line represents the HSS infusion (3M; NaCl). The arrow indicates nanoinjection in the MnPO.

The histological analysis demonstrated that saline or Muscimol nanoinjections were limited to the MnPO region (Fig. 1A). Similar RSNA, MAP, HR, baseline, body mass and blood volume withdrawn were similar in sham rats (MnPOS; n = 6) and rats that were subjected to pharmacological inhibition of MnPO (MnPOI; n = 6; Table 1). The HH-induced hypotension was similar in MnPOS (61.4 ± 0.4 mmHg) and MnPOI (62.4 ± 0.5 mmHg) after 10 min of HH, p < 0.05, Fig. 1B). At 20 min of HH, no significant change was observed in HH-induced hypotensive response that was observed at 10 min in both groups (MnPOS: 61.1 ± 0.3 mmHg *vs*. MnPOI experimental: 60.9 ± 0.4 mmHg), p > 0.05, Fig. 1B). However, sodium overload reestablished MAP baseline (96.0 ± 3.9 mmHg, 60 min after HSS infusion, Fig. 1B) in MnPOS group. However, in MnPOI rats, HSS infusion was unable to restore MAP (62.7 ± 5.0 mmHg, 60 min after HSS infusion, p < 0.05, Fig. 1B).

**Table 1:** Baseline values of body mass (g), mean arterial pressure (MAP), heart rate (bpm), integral of renal sympathetic activity (∫RSNA) and blood volume withdrawn to induced hypovolemic hemorrhage (HH) in the sham and lesioned animals. MnPOS (MnPO sham group), MnPOI (MnPO Inhibition group), A2S (A2 sham group), A2L (A2 lesioned group), A1S (A1 sham group), A1L (A1 lesioned group), A1S (A1 + A2 sham group), A1 + A2L (A1 + A2 lesioned group).

Groups	N	Body Mass (g)	MAP (mmHg)	HR (bpm)	∫RSNA (u.a)	Blood Volume (mL)
MnPOS	6	325.9 ± 7.9	102.4 ± 3.7	416.0 ± 4.5	0.09 ± 0.01	3.6 ± 0.4
MnPOI	6	318.6 ± 8.2	103.7 ± 4.7	429.3 ± 5.3	0.10 ± 0.02	3.4 ± 0.7
A2S	6	320.5 ± 5.2	108.5 ± 4.5	413.8 ± 16.4	0.10 ± 0.03	3.7 ± 0.7
A2L	6	322.5 ± 7.3	100.9 ± 2.9	431.5 ± 12.3	0.07 ± 0.02	3.9 ± 0.3
A1S	6	319.6 ± 9.1	106.2 ± 4.4	419.4 ± 6.8	0.07 ± 0.02	3.7 ± 0.6
A1L	6	320.4 ± 4.7	105.8 ± 4.5	429.7 ± 7.7	0.06 ± 0.01	4.0 ± 0.8
A1 + A2L	6	317.2 ± 6.1	111.2 ± 6.1	419.5 ± 12.6	0.10 ± 0.03	3.8 ± 0.5
A1 + A2L	6	310.6 ± 8.5	106.6 ± 3.6	428.8 ± 8.4	0.07 ± 0.01	3.6 ± 0.9

Bradycardia was observed at the end of HH in both groups (MnPOS: from 414.3 ± 4.8 to 391.7 ± 11.1 bpm *vs*. MnPOI: from 427.4 ± 5.3 to 399.0 ± 12.8 bpm after 10 min of HH, p < 0.05, Fig. 1C). Saline or muscimol nanoinjections in MnPO did not change HR (MnPOS: 371.4 ± 13.1 bpm vs. MnPOI: 376.8 ± 11.3 bpm, 20 min of HH; Fig. 1C). In both groups, HR was restored to baseline after HSS infusion (MnPOS: 392.7 ± 5.4 bpm *vs*. MnPOI: 405.4 ± 7.9 bpm, 60 min after HSS infusion; Fig. 1C).

Renal sympathoinhibition was observed during HH in MnPOS group (Δ −39.2 ± 4.3% from baseline, 10 min of HH, p < 0.05, Fig. 1D). In MnPOI there was no change in RSNA during HH (Δ −12.3 ± 5.2% from baseline, 10 min of HH, Fig. 1D). These patterns were not change by saline or muscimol nanoinjections into MnPO (MnPOS: Δ −42. ± 7.2% from baseline *vs*. MnPOI: Δ 9.3 ± 5.6 from baseline, after 20 min of HH, Fig. 1D). The sympathetic activity reduction in the renal territory of MnPOS animals was maintained after HSS infusion (Δ −45.3 ± 7.8% from baseline, 60 min after HSS infusion, p < 0.05, Fig. 1D). In contrast, sodium overload promoted renal sympathoexcitation in MnPOI group (Δ 48.0 ± 10.8% from baseline, 60 min after HSS infusion, p < 0.05, Fig. 1D).

### Effects of A2 lesion on HSS-induced cardiovascular and sympathetic responses

**Figure 2:**
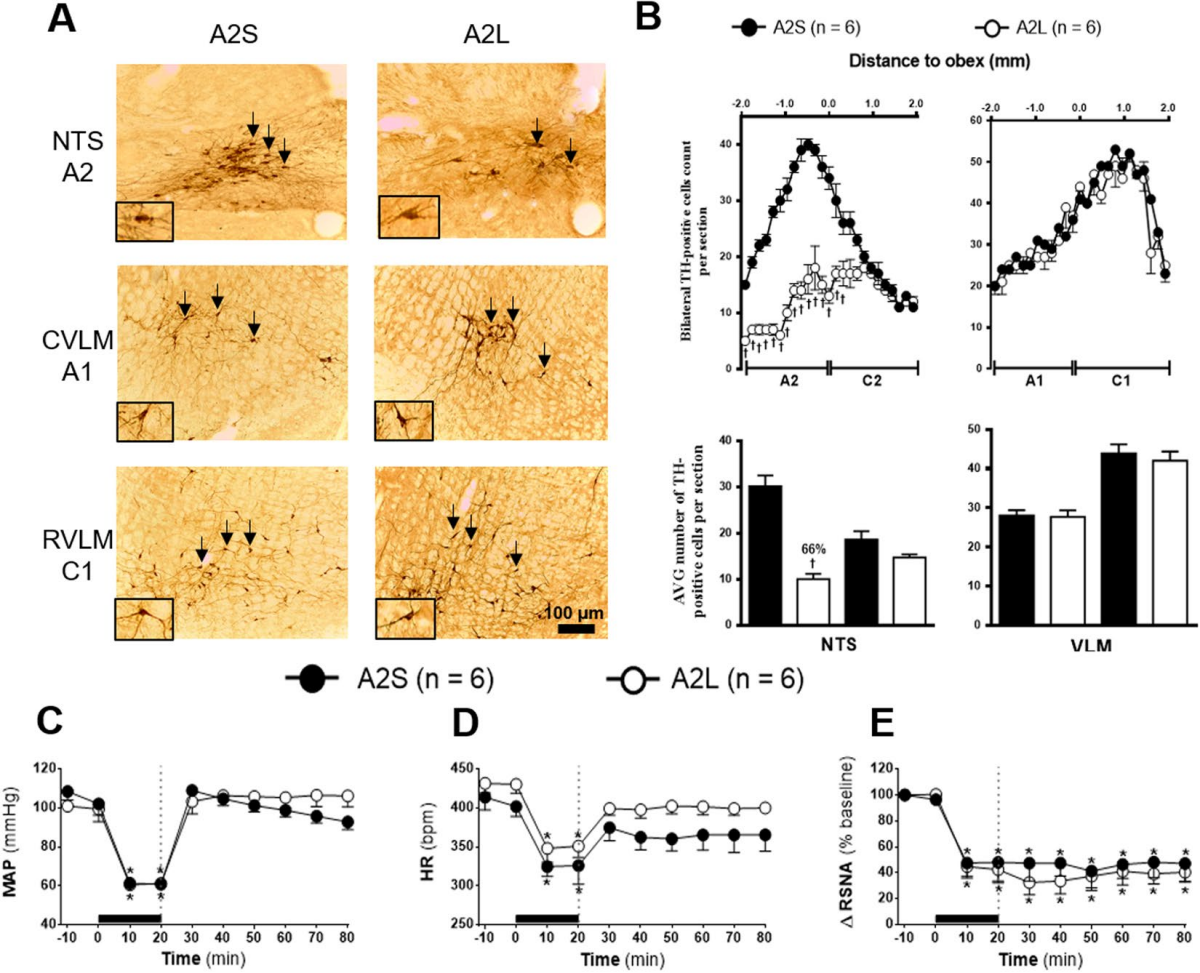
**(A)** Representative photomicrograph of coronal medulla section (40 μm) marked by immunohistochemistry. The arrows indicate the marking of TH positive neurons in the nucleus of the solitary tract (NTS - cluster A2), in caudal ventrolateral medulla (CVLM - cluster A1) and rostrovetrolateral medulla region (RVLM - cluster C1) of the animals receiving nanoinjection of bilateral saporin (A2S) or saporin-anti-DβH (A2L) in NTS. The scale bar is equal to 100 µm. **(B)** Quantification of the number of TH positive cells in the medullary regions. Mean ± standard error of the mean (S.E.M.) of the number of positive TH cells located in the nucleus of the solitary tract (NTS – clusters A2 and C2), the caudal ventrolateral medulla (CVLM – cluster A1) and rostral vetrolateral medulla region (RVLM - cluster C1) of the animals receiving nanoinjection of bilateral saporin (A2S) or saporin-anti-DβH (A2L) in NTS. ^†^Different from sham group with p < 0.05. **(C**) Effects of A2 catecholaminergic neuron lesions on changes in Mean arterial pressure (MAP), **(D)** heart rate (HR) and **(E)** integral of renal sympathetic nervous activity (∫RSNA) variations in the rats submitted to hypovolemic hemorrhagic in the A2 Sham (A2S; n = 6) and A2 lesioned (A2L; n = 6) groups. Values were expressed as means ± standard error of the mean (S.E.M). *Different from time 0; ^†^different from sham group with p < 0.05. The black bar represents the hemorrhage period. The dashed line represents HSS infusion (3M; NaCl).

**Figure 3:**
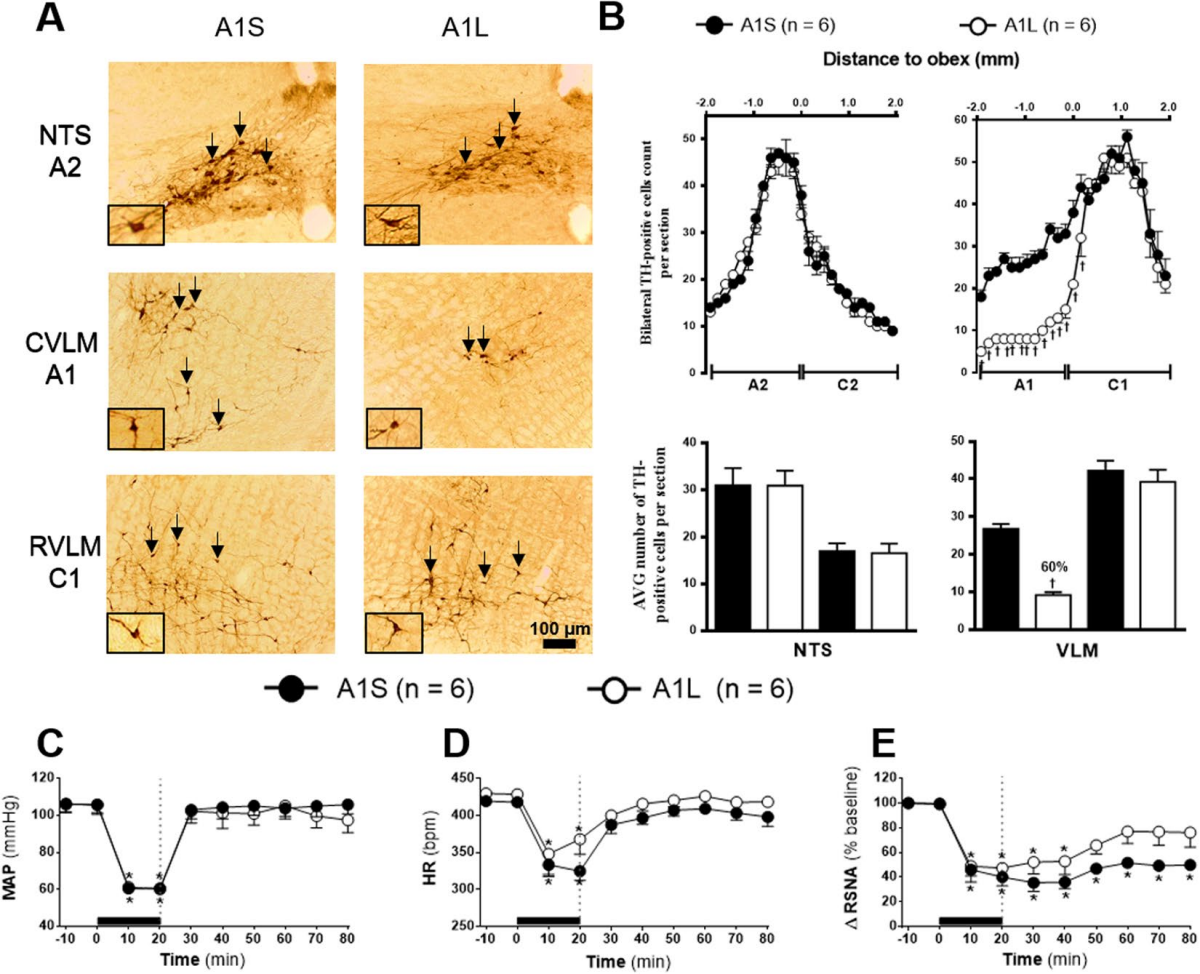
**(A)** Representative photomicrograph of coronal medulla section (40 μm) marked by immunohistochemistry. The arrows indicate the marking of TH positive neurons in the nucleus of the solitary tract (NTS - cluster A2), in caudal ventrolateral medulla (CVLM - cluster A1) and rostrovetrolateral medulla region (RVLM - cluster C1) of the animals receiving nanoinjection of bilateral saporin (A1S) or saporin-anti-DβH (A1L) in CVLM. The scale bar is equal to 100 µm. **(B)** Quantification of the number of TH positive cells in the medullary regions. Mean ± standard error of the mean (S.E.M.) of the number of positive TH cells located in the nucleus of the solitary tract (NTS – clusters A2 and C2), the caudal ventrolateral medulla (CVLM – cluster A1) and rostral vetrolateral medulla region (RVLM - cluster C1) of the animals receiving nanoinjection of bilateral saporin (A1S) or saporin-anti-DβH (A1L) in CVLM. ^†^different from sham group with p < 0.05. **(C)** Effects of A1 + A2 catecholaminergic neuron lesions on changes in Mean arterial pressure (MAP), **(D)** heart rate (HR) and **(E)** integral of renal sympathetic nervous activity (∫RSNA) variations in the rats submitted to hypovolemic hemorrhagic in the A1 Sham (A1S; n = 6) and A1 lesioned (A1L; n = 6) groups. Values were expressed as means ± standard error of the mean (S.E.M). *Different from time 0; ^†^different from sham group with p < 0.05. The black bar represents the hemorrhage period. The dashed line represents HSS infusion (3M; NaCl).

**Figure 4:**
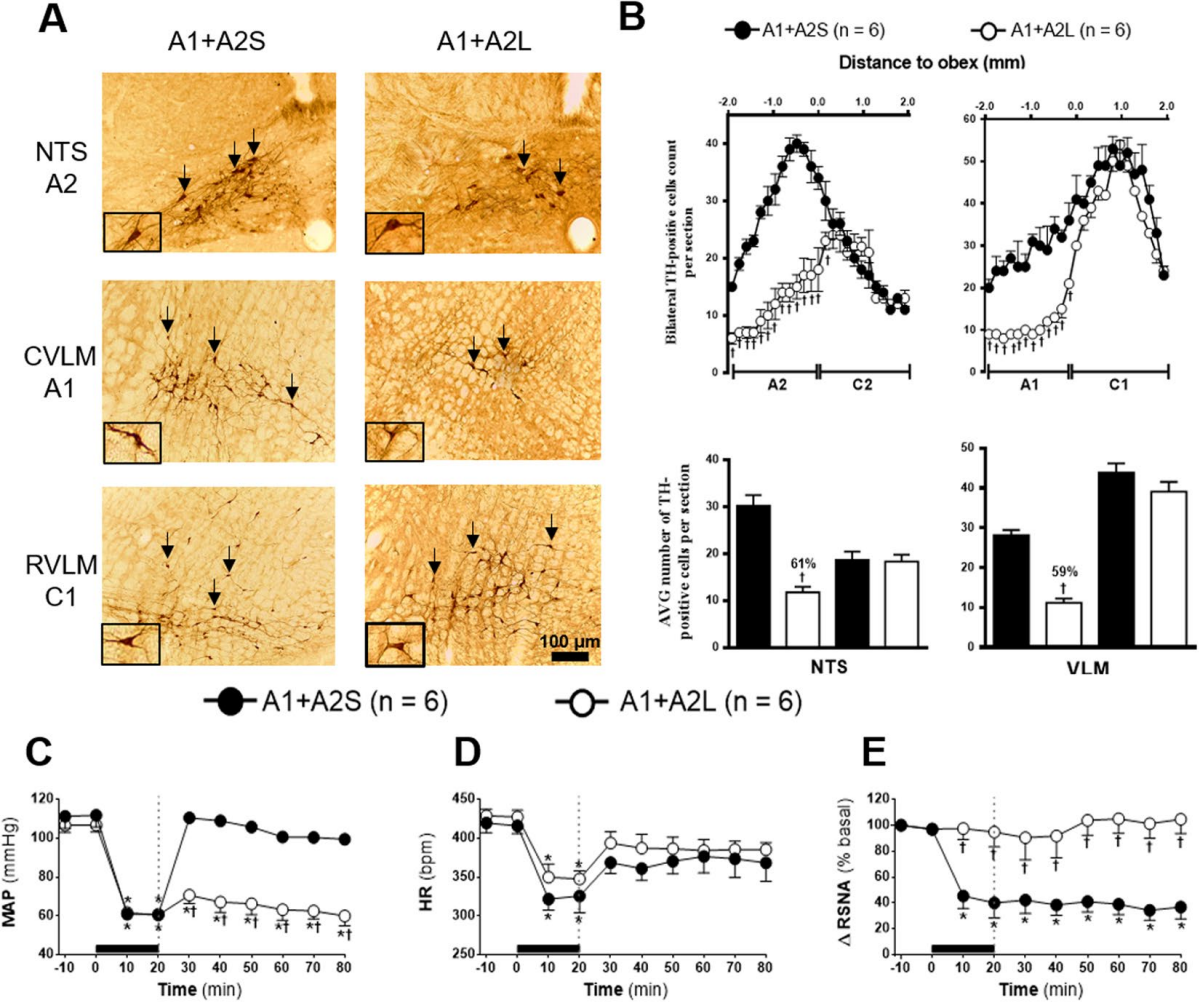
**(A)** Representative photomicrograph of coronal medulla section (40μm) marked by immunohistochemistry. The arrows indicate the marking of TH positive neurons in the nucleus of the solitary tract (NTS - cluster A2), in caudal ventrolateral medulla (CVLM - cluster A1) and rostral vetrolateral medulla region (RVLM - cluster C1) of the animals receiving nanoinjection of bilateral saporin (A1 + A2S) or saporin-anti-DβH (A1 + A2L) in CVLM and NTS. The scale bar is equal to 100 µm. **(B)** Quantification of the number of TH positive cells in the medullary regions. Mean ± standard error of the mean (S.E.M.) of the number of positive TH cells located in the nucleus of the solitary tract (NTS – clusters A2 and C2), the caudal ventrolateral medulla (CVLM – cluster A1) and rostral vetrolateral medulla region (RVLM - cluster C1) of the animals receiving nanoinjection of bilateral saporin (A1 + A2S) or saporin-anti-DβH (A1 + A2L) in CVLM and NTS. ^†^Different from sham group with p < 0.05. **(C)** Effects of A1 + A2 catecholaminergic neuron lesions on changes in Mean arterial pressure (MAP), **(D)** heart rate (HR) and **(E)** integral of renal sympathetic nervous activity (∫RSNA) variations in the rats submitted to hypovolemic hemorrhagic in the A1 + A2 Sham (A1 + A2S; n = 6) and A1 + A2 lesioned (A1 + A2L n = 6) groups. Values were expressed as means ± standard error of the mean (S.E.M). *Different from time 0; ^†^different from sham group with p < 0.05. The black bar represents the hemorrhage period. The dashed line represents HSS infusion (3M; NaCl).

Qualitative (Figs 2A, 3A, 4A) and quantitative (Figs 2B, 3B, 4B) histological analyses revealed the presence of TH positive neurons within ventrolateral medullary (VLM) and NTS extension at regions ranging from 1.900 μm caudal and 1.900 μm rostral to obex^16^.

The quantification of TH positive neurons demonstrated that A2 sham rats (A2S; n = 6) which received saporin nanoinjections into NTS have an average of 30 cells per section at the region encompassing the A2 neuronal cluster (1.900 μm caudal to obex). The average number of cells per section was reduced to 10 in A2-lesioned rats (A2L; n = 6) which received anti-DβH-saporin nanoinjections. Hence, approximately 66% of A2 neurons in the A2L group was reduced as compared to the A2S group (Fig. 2B). The anti-DβH-saporin nanoinjections in NTS did not change the number of TH positive cells at the NTS region which encompasses C2 neuronal cluster (1.900 μm rostral to the obex; Fig. 2B, 20% in relation to A2S group). No changes were observed in the number of cells at A1 (200–1.900 μm caudal to the obex and 200 μm rostral), C1 (1.900 μm caudal to the obex) and VLM (Fig. 1B, A1: −13% and C1: 11% in relation to A2S group) regions.

The RSNA baseline and blood volume that was withdrawn to promote HH were similar among groups (Table 1). The HH-induced equivalent hypotension between groups (A2S: from 108.5 ± 4.5 to 60.8 ± 0.4 mmHg *vs*. A2L: from 100.9 ± 2.9 to 61.2 ± 0, 6 mmHg, 20 min after the beginning of HH, p < 0.05, Fig. 2C). Similarly, HSS infusion reversed the hypotension generated during HH in both groups (A2S: 92.8 ± 4.0 mmHg *vs*. A2L: 106.2 ± 5.7 mmHg, 60 min after HSS infusion, Fig. 2C).

Bradycardia was observed at the end of HH in the A2S and A2L groups (A2S: from 413.8 ± 16.4 to 325.9 ± 23.9 bpm; A2L: from 431.5 ± 12.3 to 350.9 ± 14.5 bpm, 20 min after the beginning of HH, p < 0.05, Fig. 2D). After the sodium overload, HR was restored to basal levels in both groups (A2S: 365.4 ± 20.8 bpm *vs*. A2L: 400.0 ± 7.1 bpm, 60 min after HSS infusion, Fig. 2D).

In the renal territory, a similar sympathoinhibition was observed during HH in the A2 sham and A2 lesioned groups (A2S: Δ −52.0 ± 14.4 from baseline *vs*. A2L: Δ −57.6 ± 10.2% from baseline, 20 min after the beginning of HH, p < 0.05, Fig. 2E). HSS infusion did not alter the sympathoinhibition promoted by HH in both groups (A2S: Δ −52.9 ± 14.3% from baseline *vs*. A2L: Δ −59.6 ± 7.0% from baseline, 60 min after infusion of HSS, p < 0.05, Fig. 2E).

### Effects of A1 lesion on HSS-induced cardiovascular and sympathetic responses

The quantification of TH positive neurons showed that animals of A1 sham rats (A1S; n = 6), which received saporin nanoinjections into CVLM region, have an average of 30 cells per section at the VLM region encompassing the region A1 neuronal cluster (200 μm and 1.900 μm caudal to obex). The average number of cells per section was reduced to 12 in A1-lesioned rats (A1L; n = 6) which received anti-DβH-saporin nanoinjections. Hence, approximately 60% of A1 neurons in the A1L group was reduced as compared to the A1S group (Fig. 3B). The anti-DβH-saporin nanoinjections in CVLM region did not change the number of TH positive cells at the VLM region which encompasses C1 neuronal cluster (200 μm rostral and 1.900 μm caudal to obex) (Fig. 3B; 7% in relation to A1S group). No changes were observed in the number of C2 (1.900 μm rostral) and A2 (1.900 μm caudal to obex) cells of NTS (Fig. 3B, C2: 1% and A2: 5% in relation to A1S group).

Hypotension generated during HH was similar between groups (A1S: from 106.2 ± 4.4 to 60.5 ± 0.3 mmHg *vs*.A1S: from 105.8 ± 4.4 to 60.0 ± 0, 9 mmHg, 20 min after the beginning of HH, p < 0.05, Fig. 3C). After sodium overload, the MAP of A1S and A1L groups was restored similarly to basal levels (A1S: 105.8 ± 4.9 mmHg *vs*. A1L: 97.4 ± 6.6 mmHg, 60 min after HSS infusion, Fig. 3C).

At the end of HH, bradycardia was observed in both groups (A1S: from 419.4 ± 6.8 to 324.8 ± 12.7 bpm *vs*. A1L: from 429.7 ± 7.7 to 367.9 ± 20, 3 bpm, 20 min after the beginning of HH, p < 0.05, Fig. 3D). HSS infusion restored HR to baseline in A1S and A1L (A1S: 397.9 ± 12.7 bpm *vs*. A1L: 418.4 ± 7.0 bpm, 60 min after HSS infusion, Fig. 3D).

HH induced renal sympathoinhibition in both groups (A1S: Δ −60.0 ± 7.1% from baseline *vs*. A1L: Δ −52.8 ± 7.7% from baseline, 20 min after the beginning of HH, p < 0.05, Fig. 3E). Initially, HSS infusion did not change sympathoinhibition observed during HH in the A1S and A1L groups (A1S: Δ −64.1 ± 5.2% from baseline *vs*.A1L: Δ −47.2 ± 12.2% from baseline, 20 min after HSS infusion, p < 0.05, Fig. 3E). However, lately, renal sympathoinhibition was abolished in A1L group (Δ −23.9 ± 11.8% from baseline, 60 min after HSS infusion, Fig. 3E).

### Effects of A1 and A2 simultaneous lesion on HSS-induced cardiovascular and sympathetic responses

The quantification of TH positive neurons demonstrated that A1 + A2 sham rats (A1 + A2S; n = 6), which received saporin nanoinjections into CVLM and NTS regions, have an average of 28 cells per section at the CVLM region that encompassing the A1 neuronal cluster and an average 30 cells per section at the NTS region that encompasses A2 neuronal cluster. The average number of cells per section was reduced to 11 in the CVLM and 12 in the NTS in A1 + A2-lesioned rats (A1 + A2L; n = 6) which received anti-DβH-saporin nanoinjections. Hence, approximately 59% of A1 neurons and 61% of A2 neurons in the A1 + A2L group was reduced as compared to the A1 + A2S group (Fig. 4B). The anti-DβH-saporin nanoinjections in the CVLM and NTS regions did not promote significant changes in the number of TH positive cells at NTS region that encompasses C2 neuronal cluster (Fig. 4B, 2% in relation to A1 + A2S group) and in the VLM region, which includes the C1 neuronal cluster (Fig. 4B, C1: 11% as compared to A1 + A2S group).

The MAP reduction promoted by HH was similar in both groups (A1 + A2S: from 111.2 ± 6.19 to 60.7 ± 0.2 mmHg *vs*. A1 + A2L: from 106.6 ± 3.6 to 60.4 ± 0.1 mmHg, 20 min following HH, p < 0.05, Fig. 4C). HSS infusion restored MAP baseline in A1 + A2S group (99.4 ± 1.7 mmHg, 60 min after HSS infusion; Fig. 4C). However, in A1 + A2L group, sodium overload did not restore MAP (59.9 ± 5.0 mmHg, 60 min after HSS infusion, p < 0.05, Fig. 4C).

The bradycardic response generated during HH was similar between groups (A1 + A2S: from 419.5 ± 12.6 to 325.5 ± 21.5 bpm *vs*. A1 + A2L: from 428.8 ± 8.4 to 347.4 ± 10.3 bpm, 20 min after the beginning of HH, p < 0.05, Fig. 4D). Similarly, HSS infusion restored HR to basal levels in both groups (A1 + A2S: 368.3 ± 23.7 bpm *vs*. A1 + A2L: 385.1 ± 9.1 bpm, 60 min after HSS infusion, Fig. 4D).

HH caused a significant renal sympathoinhibition in A1 + A2 sham group (Δ −60.1 ± 11.5% from baseline, 20 min following HH, p < 0.05, Fig. 4E). The RSNA remains unchange by sodium overload in A1 + A2 sham group (Δ −63.2 ± 9.2% from baseline, 60 min after HSS infusion, p < 0.05, Fig. 4E). There was no change in this parameter in the A1 + A2L group during HH (Δ −5.3 ± 11.4% from baseline, 20 min after the beginning of HH, Fig. 4E) and after HSS infusion (Δ 4.6 ± 11.1% from baseline, 60 min after HSS infusion, Fig. 4E).

### Effects of A1 and A2 simultaneous neuronal lesion vs. MnPO inhibition on HSS-induced cardiovascular and sympathetic responses

**Figure 5:**
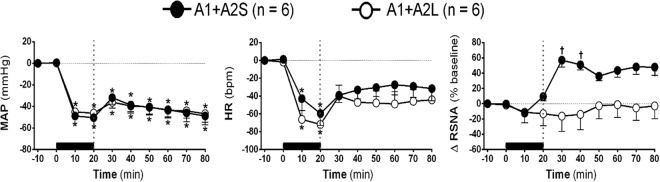
**(A)** Effects of A1 + A2 catecholaminergic neuron lesions and MnPO inhibition on changes in mean arterial pressure (Δ% MAP), **(B)** heart rate (Δ% HR) and **(C)** integral of renal sympathetic nervous activity (Δ% ∫RSNA) variations in the rats submitted to hypovolemic hemorrhagic in the A1 + A2 lesioned (A1 + A2L; n = 6) and MnPO inhibition (MnPOI; n = 6) groups. Values were expressed as means ± standard error of the mean (S.E.M.). *Different from time 0; ^†^different from A1 + A2L group with p < 0.05. The black bar represents the hemorrhage period. The dashed line represents HSS infusion (3M; NaCl).

The MAP reduction promoted by HH was similar in both groups (A1 + A2L: Δ −45.0 ± 3.9% *vs*. MnPOL: Δ −48.8 ± 2.6%, 10 min after the beginning of HH, in relation to baseline, p < 0.05, Fig. 5A). Similarly, sodium overload did not restore MAP in both groups (A1 + A2L: Δ −40.4 ± 6.6% *vs*. MnPOL: Δ −40.8 ± 7.1%, 30 after HSS infusion, in relation to baseline p < 0.05, Fig. 5A)

The bradycardic response generated during HH was similar between groups (A1 + A2S: Δ −66.2 ± 20.6% *vs*. MnPOL: Δ −42.9 ± 13.3%, 10 min after the beginning of HH, in relation to baseline, p < 0.05, Fig. 5B). Similarly, HSS infusion restored HR to basal levels in both groups (A1 + A2S: Δ −47.7 ± 13.4% *vs*. MnPOL: Δ 30.4 ± 10.3%, 30 min after HSS infusion, in relation to baseline, Fig. 5B).

The RSNA did not change after HH in the A1 + A2L and MnPOL groups (A1 + A2L: Δ −12.3 ± 5.2% *vs*. MnPOL: Δ −11.1 ± 13.8%, 10 min after the beginning of HH, in relation to baseline; Fig. 5C). The RSNA remains unchanged after sodium overload in A1 + A2L (Δ −2.7 ± 16.7% from baseline, 30 min after HSS infusion, Fig. 5C). Moreover, the inhibition of MnPO promoted simpathoexcitation (Δ 36.1 ± 5.9%, 30 min after HSS infusion, in relation to baseline, Fig. 5C).

### Effects of MnPO inhibition on baroreceptor reflexes

**Figure 6:**
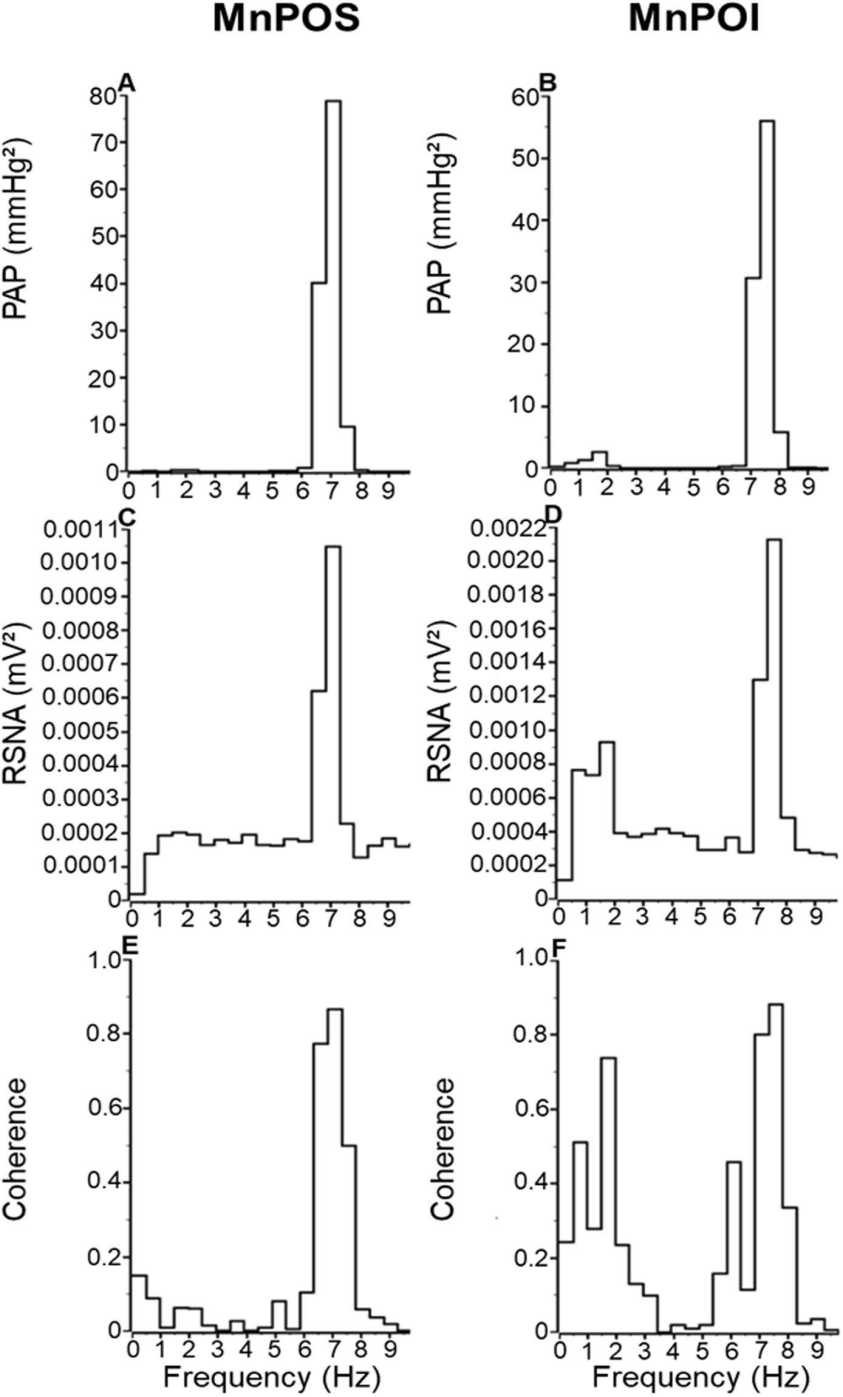
Post-systolic spectrum of **(A**,**B)** arterial pressure (AP), **(C**,**D)** renal sympathetic nerve activity (SNA) and coherence values between AP and RSNA power spectrum **(E**,**F)** in the rats submitted to hypovolemic hemorrhagic in the MnPO Sham (MnPOS; n = 6) and MnPO Inhibition (MnPOI n = 6) groups.

Baseline functionality of baroreceptor reflexes was evaluated by analysis of coherence between arterial pressure (AP) and RSNA. Similar results were observed in MnPOS and MnPOI groups. In both groups, RSNA power spectra displayed a prominent peak corresponding to the heart rate, indicating a prominent role of baroreceptor reflexes in modulating spontaneous RSNA discharge (Fig. 6). Similarly, correlation between RSNA bursts and cardiac cycle leads to high AP-RSNA coherence values (Mean - MnPOS: 0.84 ± 0.03 vs. MnPOI: 0.83 ± 0.03, Fig. 6, Table 2).

**Table 2:** Baseline values of renal blood flow (RBF), aortic blood flow (ABF), hindlimb blood flow (HBF), and coherence between arteria pressure and renal sympathetic nervous activity (Coherence AP-RSNA). MnPOS (MnPO sham group), MnPOI (MnPO Inhibition group), A2S (A2 sham group), A2L (A2 lesioned group), A1S (A1 sham group), A1L (A1 lesioned group), A1S (A1 + A2 sham group), A1 + A2L (A1 + A2 lesioned group).

Groups	N	RBF (ml·min^−1^)	ABF (ml·min^−1^)	HBF (ml.min^−1^)	Coherence AP - RSNA
MnPOS	6	2.8 ± 0.3	17.8 ± 2.4	12.2 + 1.8	0.84 ± 0.03
MnPOI	6	3.6 ± 0.7	19.1 ± 2.8	11.9 ± 1.4	0.83 ± 0.03
A2S	6	4.0 ± 0.6	54.4 ± 8.2	45.6 ± 7.5	—
A2L	6	3.5 ± 0.4	49.3 ± 3.8	42.4 ± 3.6	—
A1S	6	2.6 ± 0.3	41.5 ± 4.2	34.9 ± 4.0	—
A1L	6	3.1 ± 0.4	46.7 ± 6.8	40.5 ± 6.5	—
A1 + A2S	6	3.1 ± 0.3	45.5 ± 7.2	39.4 ± 7.7	0.87 ± 0.04
A1 + A2L	6	4.0 ± 0.3	41.9 ± 9.4	34.0 ± 9.8	0.79 ± 0.02

### Effects of lesion of A1 and A2 neuronal cluster simultaneous lesion on baroreceptor reflexes

**Figure 7:**
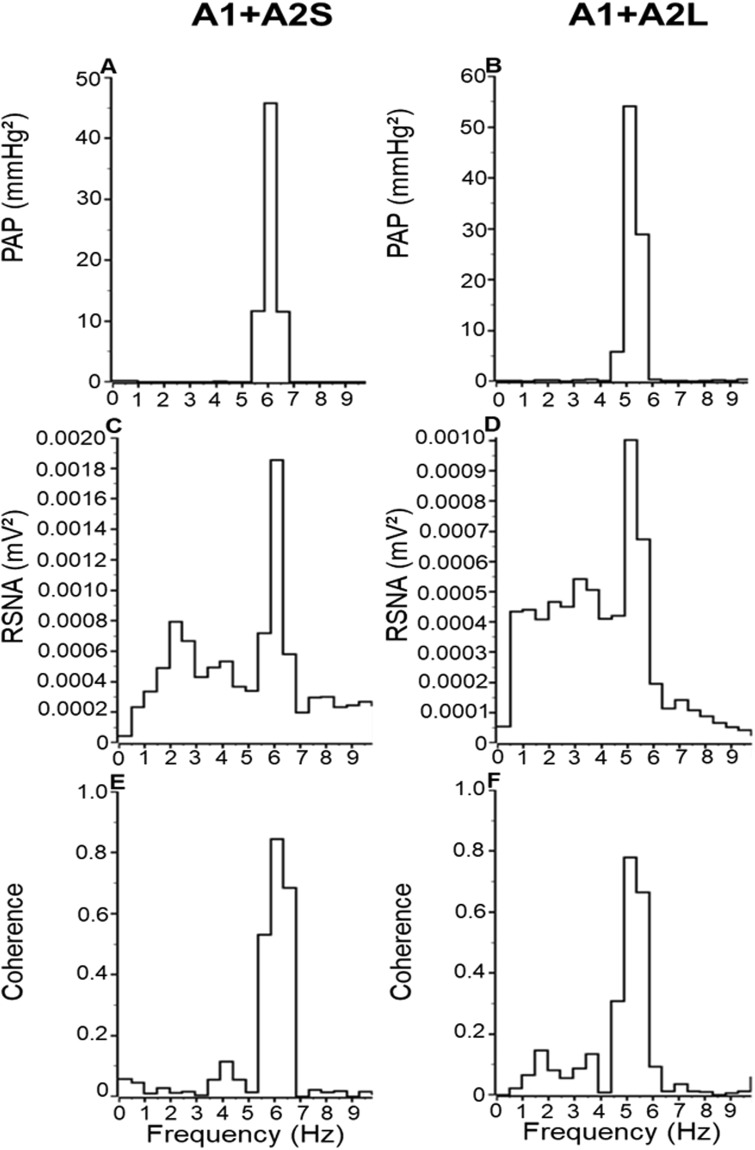
Post-systolic spectrum of **(A**,**B)** arterial pressure (AP), **(C**,**D)** renal sympathetic nerve activity (SNA) and coherence values between AP and RSNA power spectrum **(E**,**F)** in the rats submitted to hypovolemic hemorrhagic in the A1 + A2 Sham (A1 + A2S; n = 6) and A1 + A2 lesioned (A1 + A2L n = 6) groups.

In A1 + A2S or A1 + A2L animals synchronization of RSNA to the cardiac cycle was observed, a prominent peak in the power spectra corresponding to the heart rate, and high AP-SNA coherence (Mean - A1 + A2S: 0.87 ± 0.04 vs. A1 + A2L: 0.79 ± 0.02, Fig. 7, Table 2).

### Baseline values of renal, aortic e hind limb blood flow

Baseline values of RBF (MnPOS: 2.8 ± 0.3 ml·min^−1^ *vs*. MnPOI: 3.6 ± 0.7 ml·min^−1^, baseline, Table 2), ABF (MnPOS: 17.8 ± 2.4 ml·min^−1^ *vs*. MnPOI: 19.1 ± 2.8 ml·min^−1^, baseline, Table 2) and HBF (MnPOS: 12.2 ± 1.8 ml·min^−1^ *vs*. MnPOI: 11.9 ± 1.4 ml·min^−1^, baseline, Table 2) were similar in the MnPOS and MnPOI groups.

In the A2S and A2L groups the baseline RBF (A2S: 4.0 ± 0.6 ml·min^−1^ *vs*. A2L: 3.5 ± 0.4 ml·min^−1^, baseline, Table 2), ABF (A2S: 54.4 ± 8.2 ml·min^−1^ *vs*. A2L: 49.3 ± 3.8 ml·min^−1^, baseline, Table 2) and HBF (A2S: 45.6 ± 7.5 ml·min^−1^ *vs*. A2L: 42.4 ± 3.6 ml·min^−1^, baseline, Table 2) were similar.

Baseline changes were not observed in RBF (A1S: 2.6 ± 0.3 ml·min^−1^ *vs*. A1L: 3.1 ± 0.4 ml·min^−1^, baseline, Table 2), ABF (A1S: 41.5 ± 4.2 ml·min^−1^ *vs*. A1L: 46.7 ± 6.8 ml·min^−1^, baseline, Table 2) and HBF (A1S: 39.9 ± 4.0 ml·min^−1^ *vs*. A1L: 40.5 ± 6.5 ml·min^−1^, baseline, Table 2) when compared A1S and A1L animals.

In addition, there were no observable differences in baseline RBF (A1 + A2S: 3.1 ± 0.3 ml.min^−1^ *vs*. A1 + A2L: 4.0 ± 0.3 ml·min^−1^, baseline, Table 2), ABF (A1 + A2S: 45.5 ± 7.2 ml·min^−1^ *vs*. A1 + A2L: 41.9 ± 9.4 ml·min^−1^, baseline, Table 2) and HBF (A1 + A2S: 39.4 ± 7.7 ml·min^−1^ *vs*. A1 + A2L: 34.0 ± 9.8 ml·min^−1^, baseline, Table 2) when compared A1 + A2S and A1 + A2L groups.

## Discussion

Despite the establishment of cardiovascular response to HSS infusion in hemorrhagic animals^23,24^, the involvement of CNS pathways still remains unclear. In the present study, the participation of MnPO, A1 and A2 noradrenergic neurons in HSS-induced recovery during HH was demonstrated through; (I) The attenuation of MAP restoration and renal sympathoexcitation after HSS infusion and MnPO inhibition. (II) Non-alteration in the patterns of HSS-induced cardiovascular and sympathetic responses in sham rats with A1 or A2 lesion; (III) Simultaneous A1 and A2 lesion which abolished the HSS-induced MAP recovery and renal sympathoinhibition in sham rats. Taken together, these results are consistent with the view that medullary noradrenergic projection to MnPO are crucial to the development of reflex responses to the HSS-induced cardiovascular recovery during HH.

We demonstrated that there were no baseline changes in RBF, ABF and HBF in all analyzed groups. These results allow us to infer that the cardiovascular and autonomic changes observed are due to HH and HSS infusion and not due to baseline hemodynamic changes caused by previous neuronal lesions. We also demonstrated that A1 and A2 neuronal cluster simultaneous lesion and MnPO inhibition do not alter the functionality of baroreceptor reflexes. More specifically, this animals present: synchronization of RSNA to the cardiac cycle, a prominent peak in the power spectra corresponding to the heart rate, and high AP-SNA coherence. In this way, modulation of spontaneous RSNA discharge in the baseline period is mainly regulated by baroreceptor reflexes. Thus, the functionality of baroreceptor reflexes in these animals indicate once again that the results observed in the present study are not due to baseline hemodynamic changes.

Previous studies have shown that rapid blood volume withdrawal during HH could lead to reductions in venous return, HR, cardiac output, fall in blood pressure^9,25^ as well as biphasic RSNA response^26^. The activation of arterial baroreceptors mediates renal sympathoexcitation prior to vagal cardiopulmonary afferents-induced renal sympathoinhibition. Our results showed that the removal of approximately 15–25% of total blood volume promotes hypotension, bradycardia and reduction in RSNA. Thus, it is suggested that hypovolemia-induced MAP reduction is partly associated to the reduction in HR and cardiac output. The renal sympathoinhibition suggests a reduction in vascular resistance and a decrease in total peripheral vascular resistance which probably contributed to the observed hypotension.

Moreover, our data showed that the HSS infusion reestablished MAP and HR of sham rats. Several studies have reported HSS infusion-induced hemodynamic improvement after HH^9,17^. Highlighting the sympathetic component influence in the hyperosmolarity-responses, the sympathoinhibition generated in the renal territory during HH was maintained after sodium overload. Hence, these data are in agreement with studies which showed renal sympathoinhibition and vasodilation induced by high plasma sodium concentration^27–30^The renal vasodilation may be essential for the maintenance of blood flow to the kidneys.

Like in the previous study^17^, HSS infusion did not promote plasma volume expansion. Hence, volume replacement seems insufficient to explain cardiovascular hyperosmolarity-induced recovery. The chemoreceptors, baroreceptors, aortic and carotid afferents are involved in HSS-induced cardiovascular recovery^9^. In addition, experimental evidence suggests that acute increases on plasma sodium concentration could trigger central components^31^.

The role of MnPO as the control center for the electrolytic balance and integration is well established. The MnPO receives dense excitatory, mostly glutamatergic nature, projections from central osmoreceptors^32,33^. These projections includes those of organum vasculosum of the lamina terminalis (OVLT) and the subfornical organ (SFO)^32,34,35^. The electrical stimulation of SFO often lead to an increase in the frequency of action potentials of MnPO neurons^36^. In addition, this nucleus controls the release of sympathetic activity, vasopressin, thirst and salt appetite^32,37,38^. In summary, MnPO can be considered as a regulatory center of body fluids homeostasis. The pharmacological inhibition of MnPO which abolished sodium overload-induced MAP restoration after HH is consistent with the previous findings. Furthermore, we showed that MnPO is not directly involved in the HR related responses, since the inhibition of this nucleus did not alter sodium overload-induced HR restoration.

We demonstrated sympathoexcitation in the renal territory after HSS infusion and MnPO inhibition. These data are in agreement with the previous MnPO inhibition that abolished high plasma osmolarity-induced renal vasodilation^17,39^. These new data suggest involvement of MnPO in cardiovascular and autonomic responses to HSS infusion after hypovolemia.

Having established the importance of MnPO to the sodium overload-induced cardiovascular and autonomic adjustments after HH, we investigated the involvement of a specific medullary region on these responses. Considering the neuroanatomical studies that have demonstrated dense projections of the A1 and A2 neuronal clusters to MnPO^11–13^, we hypothesized A1 and A2 neurons as potentials central components that regulate the reflex adjustments induced by HSS infusion in hemorrhagic rats.

**Figure 8:**
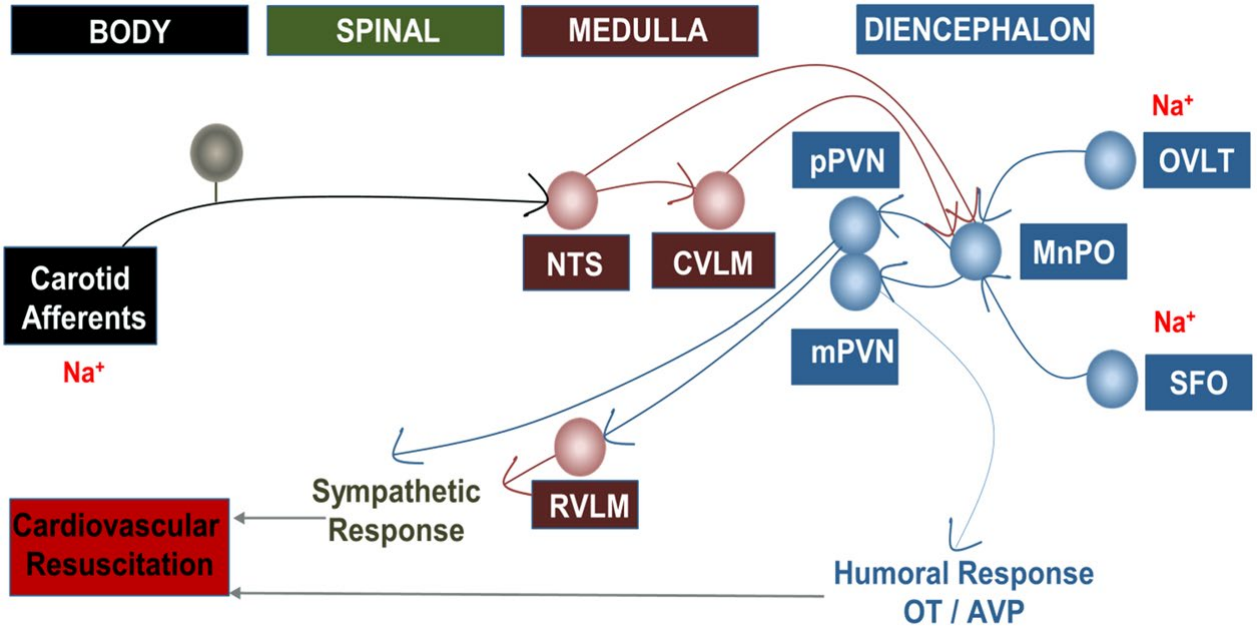
Central pathways involved in the resuscitation of hypertensive hemorrhage promoted by sodium overload. Caudal ventrolateral medulla (CVLM), nucleus of the solitary tract (NTS), magnocellular hypothalamic paraventricular nucleus (mPVN), parvocellular hypothalamic paraventricular nucleus (pPVN), median preoptic nucleus (MnPO), subfornical organ (SFO), organum vasculosum lamina terminalis (OVLT), rostrovetrolateral medulla region (RVLM), oxytocin (OT), vasopressin (VP).

The volume and composition of the circulating liquid are transmitted to the CNS through afferent fibers of peripheral sensors such as chemoreceptors, baroreceptors, cardiopulmonary receptors, and peripheral osmoreceptors^40^. In the NTS, these afferent fibers perform the first synapse and trigger a series of connections in the CNS^41^. From the NTS, A2 neuronal cluster are projected directly and indirectly, via A1 neuronal cluster, to the MnPO^11–13^. The dense projections of MnPO are sent to PVN. The PVN is a hypothalamic nucleus that regulates humoral and autonomic responses to hyperosmolarity, through vasopressin secretion and sympathetic preganglionic neurons projections, respectively^37,38^. Studies have shown that the PVN regulates the autonomic responses through its projections to the intermediate-lateral column (IML) and to the rostral ventrolateral medulla (RVLM), where sympathetic preganglionic neurons and sympathetic pre-motor neurons are located, respectively^37,40^. Thus, the integration of this extensive neural network culminates in autonomic, hemodynamic and endocrine reflexes in order to maintain body homeostasis in response to osmotic stimuli^28,30^. Within this neuronal pathway, the A1 and A2 noradrenergic neurons cluster highlight. Our studies characterize these structures as part of the fundamental pathways in the transmission of information about sodium overload-induced changes in plasma osmolarity after HH (Fig. 8).

Although, the lesion of A1 or A2 neuronal clusters did not alter sodium overload -induced cardiovascular and autonomic responses after hypovolemia, we cannot exclude central parallel pathways which regulate same function. The latter assumption is supported by the previous studies which demonstrated that concomitant lesion, not the individual lesion, of AV3V region and NTS reduced MAP in spontaneously hypertensive rats^42^.This investigation demonstrated the importance of parallel pathways to cardiovascular control. In this sense, concomitant lesion of A1 and A2 neuronal cluster could cause complete loss of compensatory mechanisms. It is important to note that A1 and A2 neuronal clusters have the same embryological origin, similar projections to the hypothalamic regions and similar responses to hyperosmotic stimuli. Hence, it is possible that these clusters possess parallel functional pathways. In order to test this conceptual model, we show that concomitant lesion of A1 and A2 neuronal groups abolished compensatory mechanism, HSS-induced MAP restoration and renal sympathoinhibition after HH.

For the first time, we showed that simultaneous lesion of A1 and A2 neurons or MnPO inhibition attenuated HSS-induced MAP restoration and renal sympathoinhibition after HH. These data show the involvement of CNS components in the regulation of these reflex responses. Thus, the integrity of MnPO, A1 and A2 neuronal cluster seems to be important to the communication between peripheral afferents and central structures.

## Conclusion

Taken together, our findings strengthen the hypothesis that the MnPO, A1 and A2 are part of the pathways that integrate and transmit information regarding changes in plasma osmolarity as well as modulating HSS-induced cardiovascular and autonomic responses after hypovolemia. The dysfunctions of MnPO, A1 and A2 could impair HSS-induced cardiovascular recovery after HH.
